# Telomere length measurement can distinguish pathogenic from non-pathogenic variants in the shelterin component, TIN2

**DOI:** 10.1111/j.1399-0004.2010.01605.x

**Published:** 2012-01

**Authors:** T Vulliamy, R Beswick, MJ Kirwan, U Hossain, AJ Walne, I Dokal

**Affiliations:** Centre for Paediatrics, Blizard Institute of Cell and Molecular Science, Barts and The London School of Medicine and Dentistry, Queen Mary University of LondonLondon, UK

**Keywords:** bone marrow failure, dyskeratosis congenita, shelterin, telomere length

## Abstract

Dyskeratosis congenita (DC) is a heterogeneous bone marrow failure syndrome with seven disease-causing genes identified to date, six of which are linked to telomere maintenance. Mutations in one of these genes (*TINF2*), which encodes a component of the shelterin complex, are associated with particularly short telomeres. Among the 224 consecutive patients with different forms of bone marrow failure (46 with DC, 122 with aplastic anaemia and 57 with some features of DC), we have identified 16 new families with variants in exon 6 of the *TINF2* gene, eight of which are novel. We observe that the phenotype associated with these mutations extends to a severe early presentation, not always classified as DC. In addition, we see that some of the variants identified are not associated with short telomeres and are also found in asymptomatic individuals. In the absence of any direct functional assay, the data indicates that the telomere length measurement can inform us as to which variants in *TINF2* are pathogenic and which may be non-pathogenic.

**Conflict of interest:**

All authors report no potential conflicts of interest.

Dyskeratosis congenita (DC) is a heterogeneous disorder, both genetically and phenotypically (1). Clinical presentation is classically defined by a triad of mucocutaneous features: abnormal reticulate skin pigmentation, nail dystrophy and leukoplakia. Bone marrow failure and a spectrum of other somatic abnormalities are also commonly observed (2). In its severe form, DC overlaps with the Hoyeraal–Hreidarsson (HH) syndrome, characterized by immunodeficiency, cerebellar hypoplasia, microcephaly and growth retardation as well as aplastic anaemia (AA). Because of this wide range of phenotypes, a clinical diagnosis of DC can often be quite difficult, although we would usually define a DC patient as having at least two of the three mucocutaneous features with either evidence of bone marrow failure or two or more other somatic abnormalities (3). In some cases a genetic diagnosis can be made, based on the identification of a mutation in one of the seven genes (*DKC1*, *TERC*, *TERT*, *NOP10*, *NHP2*, *TINF2* and *C16orf57*).

Through genome-wide linkage analysis and candidate gene sequencing, the *TINF2* gene was initially identified as the cause of DC in one family showing autosomal dominant inheritance of the disease (4). It has subsequently become clear that mutations in this gene usually arise *de novo* in sporadic cases, causing a relatively severe form of DC (4, 5). The gene encodes a core component of the shelterin complex – a group of proteins that interact to protect telomeres. This protein is called TIN2, the telomeric repeat binding factor 1 (TRF1)-interacting nuclear factor 2; it is an essential mediator of TRF1 function and acts as an important regulator of telomere length (6, 7). TIN2 acts as the central component of the shelterin complex, binding not only TRF1 but also TRF2, a second telomere DNA-binding protein (8) and TPP1, the TIN2-interacting protein (9). TIN2-anchored TPP1 plays a major role in the recruitment of telomerase to telomeres in human cells (10). TPP1 is also important for recruiting POT1 (protection of telomeres), which is the third DNA-binding protein of the shelterin complex. POT1 binds to telomeric single-stranded DNA, protecting chromosome ends from the DNA-damage response (11). A second larger isoform of TIN2 has recently been identified, and this appears to have a role in tethering telomeres to the nuclear matrix (12).

Since our previous report in 2008 (5), we have been screening for *TINF2* mutations in all patients referred to our DC registry with various forms of bone marrow failure. This has led to the identification of 16 new families with eight previously unreported variants. They show that the phenotype associated with *TINF2* mutation is broader than previously thought, but also raise the question as to whether all of these novel variants are pathogenic.

## Subjects and methods

Informed consent was obtained for all samples referred to the DC registry. The subjects included in this study were collected during a 2-year period following our previous report on the role of TIN2 mutations in DC (5). They comprise 45 new unrelated cases classified as having DC or HH based on the clinical criteria described above. In addition, we have screened 122 subjects with idiopathic AA and 57 subjects who had apparently constitutional AA, or had disease features overlapping those of DC. Whenever a variant in the *TINF2* gene was identified, we proceeded to screen all available family members.

DNA was extracted from whole blood using the Puregene DNA isolation kit (Gentra, Qiagen, Crawley, UK). We have targeted our mutation screen to exon 6 of the *TINF2* gene, as this is where all pathogenic mutations have been shown to lie in a cohort of 131 DC patients (5). A 618 bp fragment extending from c.605-74 to c.1061+87 was amplified using the primers 5′GGCTCCGGG CATAAGAAAC3′ and 5′TGAGGTGAGAGCAA GCAAAG3′ (Sigma-Genosys, Poole, UK) in a PCR (Thermo Fisher Scientific, Runcorn, UK). These fragments were then analysed by denaturing high-performance liquid chromatography (Trans genomic, Glasgow, UK): where abnormal elution patterns were identified, the fragment was then re-amplified and sequenced directly using BigDye terminators (Applied Biosystems, Warrington, UK) after clean-up using exonuclease and shrimp alkaline phosphatase (ExoSap, GE Healthcare, Little Chalfont, UK).

Telomere lengths were measured by Southern blot analysis using a subtelomeric probe from the short arm of chromosome 7 as previously described (13).

## Results

Over the 2-year period since our previous report on *TINF2* mutations in patients with DC (5), we have identified 7 new patients with heterozygous *TINF2* variants out of 46 patients who were classified as having DC or HH on clinical criteria. Of these, five have the Arg282His substitution, one has the Arg282Cys substitution and one has a novel mutation that results in a Thr284Lys substitution (Table 1). Both of the mutations to the Arg282 residue have been reported previously as disease causing and are seen to occur repetitively among patients who have a relatively severe form of the disease (4, 5, 14). For five of the six cases with these Arg282 substitutions, we have samples from both parents and in each case neither parent was found to carry the mutation. This is consistent with previous observations that the majority of disease-causing *TINF2* mutations arise *de novo.* For the patient with the Thr284Lys substitution, we surprisingly find that his father and sister both have a different mutation affecting the same residue (resulting in a Thr284Ile substitution) which also has not been previously reported.

**Table 1:** New patients with mutations in the shelterin component, TIN2

ID	Age/sex	Clinical features^a^	Nucleotide substitution^b^	Amino acid substitution^b^	Δtel^c^
Dyskeratosis congenita					
D1	9/F	Skin, nails, BMF, epiphora, short stature	c.845G>A	p.Arg282His	−3.07
D2	4/F	Skin, nails, BMF, leukoplakia, dental abnormalities	c.845G>A	p.Arg282His	−5.24
D3	11/F	Skin, nails, BMF, dental caries/loss	c.844C>T	p.Arg282Cys	n/a^d^
D4	5/M	Skin, nails, BMF, leukoplakia, epiphora, esophageal stricture	c.845G>A	p.Arg282His	n/a
D5	14/M	Skin, nails, BMF	**c.851C>A**^e^	**p.Thr284Lys**	**n/a**
Hoyeraal–Hreidarsson syndrome					
H1	1/M	BMF, growth retardation, Coat's retinopathy, intracranial calcification, cerebellar hypoplasia, ataxia	c.845G>A	p.Arg282His	n/a
H2	2/F	BMF, microcephaly, ataxia, cerebellar hypoplasia, developmental delay	c.845G>A	p.Arg282His	−5.37
With features overlapping dyskeratosis congenita					
V1	3/M	BMF (familial)	**c.640C>T**	**p.Pro214Ser**	**n/a**
V2	4/M	Nails, BMF	c.845G>A	p.Arg282His	−8.23
V3	5/M	Nails, BMF, low BW, hiderosis, clinodactyly, intracranial calcifications	c.845G>A	p.Arg282His	n/a
V4	4/M	Mucocutaneous features; BMF	**c.805C>T**	**p.Gln269X**	**−6.53**
V5	7/F	Skin, BMF, small face, epicanthic folds	c.734C>A	p.Ser245Tyr	+1.33
V6	3/M	Nails, BMF, microcephaly, low immunoglobulins	**c.857delTinsGC**	**p.Met286SerfsX5**	**n/a**
V7	3/F	Nails, BMF, lichenoid tongue, dry skin, IUGR	**c.826delA**	**p.Arg276GlyfsX41**	**−3.80**
V8	66/F	BMF, hair loss, dental loss, pulmonary disease, short stature, osteoporosis	**c.851C>G**	**p.Thr284Arg**	**n/a**
V9	3/F	Nails, BMF	**c.849delC**	**p.Thr284GlnfsX33**	**n/a**
Relatives					
R1	45/M	None, father of D5	**c.851C>T**^f^	**p.Thr284Ile**	**+0.65**
R2	40/M	Vitiligo, father of V1	c.640C>T	p.Pro214Ser	n/a
R3	35/F	None, mother of V5	c.734C>A	p.Ser245Tyr	+1.34

BMF, bone marrow failure; BW, birth weight; F, female; IUGR, intrauterine growth retardation; M, male.

a Skin, abnormal skin pigmentation; nails; nail dystrophy.

b Nucleotide and amino acid substitutions are numbered according to Human Genome Variome Society guidelines.

c The difference between the observed telomere length and the telomere length predicted according to age (20).

d Not available because of insufficient/good quality DNA.

e Mutations in bold type have not been reported previously.

f The father of patient D5 has a mutation that is different from that of his affected son.

Over the same period, we have also been looking for *TINF2* mutations among patients who have bone marrow failure but do not have the clinical hallmarks of DC: this series comprised 122 patients with AA and 57 patients in whom there are some features that overlap DC. Among this group, we have identified an additional nine patients with variants in exon 6 of the *TINF2* gene. Of these, only two have Arg282His, one has the previously described Ser245Tyr substitution (5), and six have previously unreported mutations (Table 1). Most of these patients have features that closely overlap DC, but which were not considered sufficient to be entered into the registry. This is probably because of the very early onset of disease in these individuals, reinforcing the suggestion that mutations to this component of the shelterin complex are associated with a severe phenotype.

**Fig. 1:**
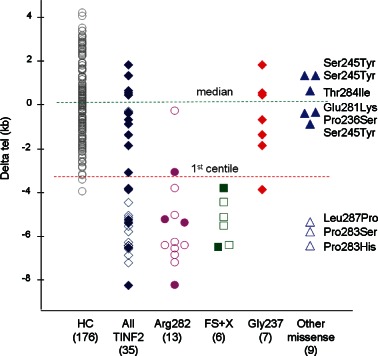
Telomere length measurement in subjects with *TINF2* mutations. Age-adjusted Δtel measurements from Southern blot analysis. A comparison is made among all subjects with *TINF2* missense mutations (dark blue diamonds), those with Arg282 substitutions (pink circles), frameshift and nonsense mutations (green squares), the polymorphic Gly237Asp substitution (red diamonds) and other missense mutations (blue triangles). Data points with open symbols are from a previous study (5), while data points obtained in the current study are shown as filled symbols. Also shown are the median (green dotted line) and the 1st centile (red dotted line) of the normal range, established from 176 healthy controls (HC, grey circles).

It is well known that patients with DC have short telomeres (15–17) and in fact they appear to be exceptionally short in cases with *TINF2* mutations (5). We have therefore measured telomere lengths by Southern blot analysis in all of the mutated patients and relatives in this study from whom we have sufficient DNA (*n* = 9), as well as a small group of previously published non-DC patients (*n* = 3) with the TIN2 substitutions Pro236Ser, Ser245Tyr and Glu281Lys. In addition, during the course of our screening experiments we have identified patients and relatives with the polymorphic Gly237Asp substitution of TIN2 (rs17102313) and have measured telomere lengths in these individuals (*n* = 7). When we combine these results with the data from our previously reported DC patients (5) (*n* = 16), what emerges is an apparently bimodal distribution of telomere length (Fig. 1).

As expected, of those with Arg282His, Arg282 Cys, nonsense and frameshift mutations, all except one have very short telomeres. In contrast, of those with the polymorphic Gly237Asp substitution, all except one have telomere lengths within the normal range (Fig. 1). However, the patients with alternative missense mutations clearly fall into two groups (Fig. 1): those with very short telomeres (Pro283His, Pro283Ser and Leu287Pro) and those with normal telomere lengths (Pro236Ser, Ser245Tyr, Glu281Lys and Thr284Ile).

**Fig. 2:**
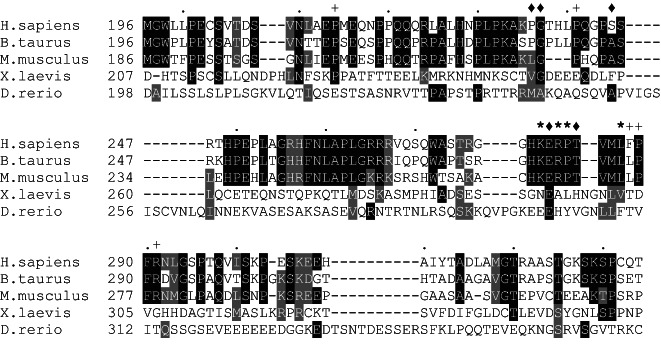
Alignment of vertebrate TIN2 protein sequences. Alignment was obtained using the clustal 2.0.12 multiple sequence alignment program and printed in boxshade. Residues are highlighted if more than 50% are identical (white on black) or similar (white on grey) to each other at any one position. The region shown extends from the beginning of the telomeric repeat binding factor 1-binding domain of human TIN2 (residues 196–275), and finishes at human residue 334. Amino acids found to be substituted in patients with dyskeratosis congenita and their relatives are highlighted as follows: those associated with normal telomere lengths are shown with a black diamond, those associated with short telomeres are shown with a bold asterisk, and those with unknown telomere lengths shown with a + sign. Every 10th amino acid is highlighted by a dot.

None of these alternative missense mutations were reported in a screen of 298 healthy control individuals (4) or listed in the latest (May 2010) release from the 1000 Genomes project. Using the prediction tools PolyPhen (polymorphism phenotyping) and sift (sorts intolerant from tolerant) to assess the impact of these mutations, we did not clearly separate the two groups as defined by telomere length (Table 2). PolyPhen predicted that of the three alternative missense mutations with very short telomeres, two were ‘probably damaging’ and one was ‘possibly damaging’ while of the four alternative missense mutations with normal telomere lengths, two were predicted to be ‘possibly damaging’ while two were ‘benign’. sift indicated that only one of these mutations is tolerated (Pro236Ser). The extent to which the substituted amino acids are conserved across species is again variable and again does not clearly distinguish the two groups (Fig. 2, Table 2).

**Table 2:** Predicted effects and degree of conservation of all known *TINF2* missense mutations

Substitution	PolyPhen prediction	sift prediction	Conservation^a^	Telomere length (Δtel)^b^	Reference
Pro214Ser	Probably damaging	Not tolerated	B, M, F	n/a	This article
Pro236Ser	Possibly damaging	Tolerated	B	Normal (−0.40)	(5)
Gly237Asp	Possibly damaging	Not tolerated	B, M, F	Normal (median = −0.70, *n* = 7)	rs17102313
Pro241Ser	Probably damaging	Not tolerated	B, M	n/a	rs17102311
Ser245Tyr	Benign	Not tolerated	None	Normal (mean = 0.60, *n* = 3)	(5); this article
Lys280Glu	Benign	Not tolerated	B, M	Very short (n/a)	(4)
Glu281Lys	Benign	Not tolerated	B, M, F, Z	Normal (−0.34)	(5)
Arg282Cys	Probably damaging	Not tolerated	B, M	Very short (mean = −5.03, *n* = 2)^c^	(5)
Arg282His	Possibly damaging	Not tolerated	B, M	Very short (median = −6.43, *n* = 10)	(4)
Arg282Ser	Possibly damaging	Not tolerated	B, M	Very short (n/a)	(4)
Pro283Ala	Probably damaging	Not tolerated	B, M	n/a	(5)
Pro283His	Probably damaging	Not tolerated	B, M	Very short (−6.40)	(5, 14)
Pro283Ser	Probably damaging	Not tolerated	B, M	Very short (−5.77)	(5)
Thr284Ala	Benign	Not tolerated	B, M	n/a	(5)
Thr284Arg	Possibly damaging	Not tolerated	B, M	n/a	This article
Thr284Ile	Possibly damaging	Not tolerated	B, M	Normal (0.65)	This article
Thr284Lys	Possibly damaging	Not tolerated	B, M	n/a	This article
Leu287Pro	Possibly damaging	Not tolerated	B, M, Z	Very short (−5.33)	(5)
Phe288Leu	Benign	Not tolerated	Z	n/a	(9)
Pro289Ser	Probably damaging	Not tolerated	B, M	n/a	(5)
Arg291Gly	Probably damaging	Not tolerated	B, M	n/a	(5)

PolyPhen, polymorphism phenotyping; sift, sorts intolerant from tolerant substitutions.

a Conservation, as determined by Clustalw alignment of the human amino acid sequence (Q9BSI4), with sequences of the B, bovine (A7E3R3); M, mouse (Q9QXG9); F, frog (A0JMZ2); Z, zebrafish (A1A5W1) orthologues.

b The difference between the observed telomere length and the telomere length predicted according to age (20).

c A third patient with the Arg282Cys substitution is very unusual in having normal telomere length (5).

## Discussion

In this study we report on 16 new families in which we have identified mutations in the *TINF2* gene, eight of which are novel. We see that the phenotype associated with these mutations extends to a severe early presentation, not always classified as DC. We also find that some *TINF2* mutations can be present in asymptomatic individuals, and so the question arises: are all of these different mutations pathogenic? It is noticeable that among the ‘non-DC’ patients in this study, there is a greater diversity in the variants identified. In addition to Arg282His, which is known to be disease causing, it is likely that mutations that cause nonsense or frameshift mutations in this crucial region of the protein are also pathogenic. But for the remaining missense mutations, we have to be more careful as these may or may not be disease causing (18). In a previous study (14), 6 out of 109 paediatric patients with severe AA were found to have *TINF2* mutations, four of which affected Arg282 and one was a frameshift mutation, presumed to be pathogenic. For the remaining missense mutation (Phe288Leu), it was unclear whether the substitution should be regarded as deleterious. More recently, 2 of 142 Japanese patients diagnosed with acquired AA or refractory anaemia were found to have TINF2 mutations (19), one of which is a previously described missense mutation (Pro283His) and one is a novel frameshift mutation. Both were associated with short telomeres, and in each case there were no physical features of DC or HH.

Of the subjects reported here who have missense mutations associated with normal telomere lengths, none present with a classical or severe DC phenotype. The index case with the Ser245Tyr substitution is a young girl with bone marrow failure and other somatic abnormalities; she has an asymptomatic mother who is also heterozygous for the mutation. The same mutation has been observed previously in a 50-year-old with AA (5). The novel Thr284Ile substitution is found only in the asymptomatic father and sister of a patient with DC. A previously reported patient (5) with the Glu281Lys mutation is unusual in that he presented only with a low white blood cell count at the age of 30.

We therefore suggest that telomere length measurement may be able to distinguish the mutations that cause disease from those that are polymorphic or bystander mutations. In the absence of any direct functional assay of these mutations, this can inform us as to the pathogenic nature of a novel missense mutation in *TINF2*.

It is unfortunate that for a significant number of patients we only have small amounts of DNA available – insufficient for telomere length measurement by Southern blot analysis. Nevertheless, consistent with previous findings, we see that all patients with missense mutations to the Arg282 residue or frameshift or nonsense mutations in this region of TIN2 have very short telomeres. These mutations often occur *de novo* and are pathogenic. The other missense mutations in this region of the gene can be divided into two groups based on telomere length measurement: those that are associated with very short telomeres, which can be assumed to be disease causing and those that are associated with normal telomere lengths, which may not be pathogenic.
